# Editorial: Exploiting genetics and genomics to improve the understanding of eye diseases

**DOI:** 10.3389/fgene.2023.1308071

**Published:** 2023-10-18

**Authors:** Denis Plotnikov, Jeremy A. Guggenheim, Xiaoyi Raymond Gao

**Affiliations:** ^1^ Central Research Laboratory, Kazan State Medical University, Kazan, Russia; ^2^ School of Optometry and Vision Sciences, Cardiff University, Cardiff, United Kingdom; ^3^ Department of Ophthalmology and Visual Sciences, The Ohio State University, Columbus, OH, United States; ^4^ Department of Biomedical Informatics, The Ohio State University, Columbus, OH, United States; ^5^ Division of Human Genetics, The Ohio State University, Columbus, OH, United States

**Keywords:** genetics, genomics, GWAS, ophthalmology, genes

Eye diseases impose a significant global health burden, affecting millions across diverse demographics. Common conditions such as age-related macular degeneration, glaucoma, and diabetic retinopathy, along with less prevalent hereditary disorders like retinitis pigmentosa and congenital cataracts, impair vision and impact quality of life and productivity. Advances in genetics and genomics have transformed the landscape of eye disease research. Traditional diagnostic methods and treatments are being augmented, and in some cases, replaced, by cutting-edge genomic technologies. Genomic research has not only facilitated the identification of disease-associated genetic variants but has also paved the way for innovative therapeutic approaches, such as gene editing and gene therapies. This Research Topic provides an insight into how genetics and genomics are reshaping the field of ophthalmology, offering new avenues for understanding, diagnosing, and treating eye diseases.

The articles in this Research Topic can be broadly classified in following groups: A review article summarizing recent progress in genome-wide association studies of intraocular pressure, two high-quality case reports, and a series of cutting-edge research articles covering a wide range of topics.

Glaucoma remains a leading cause of global irreversible blindness. In the review article by Gao et al. the focus was on intraocular pressure (IOP), which is the only known modifiable risk factor for glaucoma. Genome-wide association studies (GWASs), primarily conducted in European and Asian ancestries, have revealed over 190 genetic loci associated with IOP. Most of these loci were identified through common variants. These findings have led to the development of polygenic scores (PGS) for predicting IOP and glaucoma risk. Recent large-scale exome-wide association studies (ExWAS) have successfully identified rare variants in 40 novel genes, some being noteworthy drug targets for clinical treatment. However, since the majority of GWASs have been conducted in individuals of European descent, limited information exists currently about underrepresented populations such as Latinos and Africans. Current PGSs perform very well in European populations, but the PGS “portability gap” has the potential to create or worsen existing health disparities. Efforts to increase diversity in GWASs, such as the All of Us research program in the United States, aim to address this imbalance. The future of IOP GWASs may involve whole-genome sequencing approaches, aided by advancements in artificial intelligence, leading to personalized genetic applications in prevention, diagnosis, and treatment across diverse patient backgrounds.

In the first of the two case reports in this Research Topic, Chen and Lu report on the AB variant GM2 gangliosidosis, an exceptionally rare autosomal recessive lysosomal storage disease, described in a 7-month-old infant from China. The child presented with nystagmus, and ophthalmic examinations revealed a distinctive cherry-red spot surrounded by a whitish infiltrate around the macula. Optical coherence tomography (OCT) revealed abnormal thickening and increased reflectivity in inner retinal layers. Genetic testing confirmed a homozygous deletion in the *GM2A* gene’s exon 2, leading to the diagnosis of AB variant GM2 gangliosidosis. This case, the first reported in China, underscores the pivotal role of ophthalmic examinations in early disease detection. The cherry-red spots observed were distinct from those caused by central retinal artery occlusion and were accompanied by unique OCT findings, providing valuable clinical insights into this rare disease’s ocular manifestations. In the case report by Peng et al., a novel frameshift variant (c.394delG, p.V132Sfs*15) within the *CRYGC* gene was identified in a patient with congenital cataract, contributing to our understanding of the genetic basis of congenital cataracts in the Chinese population. The research utilized whole-exome sequencing to pinpoint the genetic defect in a patient lacking a family history of the condition. This rapid and accurate genetic diagnosis proved invaluable in guiding clinical decisions and ensuring timely intervention, highlighting the importance of genetic testing in pediatric cases. The identified variant disrupted a critical region of the *CRYGC* gene, shedding light on the structural changes responsible for congenital cataracts. This study adds to the growing body of knowledge about the genetic diversity underlying cataract disorders and emphasizes the significance of precise genetic diagnoses in pediatric ophthalmology.

Two brief research reports in this Research Topic highlighted the role, first, of DNA methylation in microRNA (miRNA) genes in patients with high myopia (HM), and, second, the role of the polymorphisms rs2472493 in *ABCA1* and rs7636836 in *FNDC3B* in pseudoexfoliation glaucoma (PXG) and primary angle-closure glaucoma (PACG). Swierkowska et al. observed specific methylation patterns in miRNA genes associated with HM. The study highlighted differential methylation levels in CG dinucleotides located in the promoter regions of several miRNA genes, including *MIR3621*, *MIR34C*, *MIR423* (with increased methylation) and *MIR1178*, *MIRLET7A2*, *MIR885*, *MIR548I3*, *MIR6854*, *MIR675*, *MIRLET7C*, *MIR99A* (with reduced methylation). These findings implicate potential disruption in the regulation of miRNA expression in the pathogenesis of HM. Additionally, the study linked these differentially-methylated miRNA genes to target genes associated with eye-related biological pathways, providing insights into the molecular mechanisms underlying HM. While the study was conducted on blood samples, it offers promising directions for further research into non-invasive diagnostic biomarkers for HM. Kondkar et al. reported an in-depth analysis of two gene polymorphisms, rs2472493 in *ABCA1* and rs7636836 in *FNDC3B*, previously linked to primary open-angle glaucoma (POAG). The study included 442 subjects, comprising healthy controls, and patients with PACG or PXG. While overall associations were not significant, the rs7636836[T] allele was associated with an increased risk of PXG in males. Additionally, a combination of alleles (G-T) was associated with increased PACG risk. Further research in larger, more diverse cohorts would help to clarify the clinical impact of these findings and pave the way for additional gene-gene and gene-environment interaction analyses in patients with glaucoma.

Eight original research papers employed diverse methods to offer new insights into the development of different eye conditions. A GWAS of color vision defects in isolated Silk Road communities, conducted by Nardone et al., identified potential genetic associations for both Deutan-Protan (DP) and Tritan (TR) traits. For DP, the genes *PIWIL4*, *MBD2*, and *NTN1* were highlighted as candidates linked to retinal health and visual signal transmission. TR traits were associated with the *VPS54*, *IQGAP1*, *NMB*, and *MC5R* genes, highlighting potential roles in RPE regulation, cone imbalances, and lacrimal gland function. In their extensive study, Jiang et al. conducted a GWAS in a sample of 19,420 adults from diverse ethnic backgrounds to explore the genetic underpinnings of ocular axial length (AL), a clinical feature of myopia. They identified 16 genomic loci associated with AL, including five novel discoveries. The loci associated with AL were also significantly associated with spherical equivalent refractive error (SER) and myopia; moreover, genetic correlations between AL, SER, and myopia were found, suggesting shared genetic etiology. The study highlighted potential candidate genes such as *SLC25A12*, *BMP3*, *RGR*, *RBFOX1*, and *MYO5B*, shedding light on their roles in visual function and eye development. The research also unveiled a genome-wide association at the *PRSS56* gene locus, a gene associated with ocular axial growth, in individuals of European ancestry. While the study focused only on common variants, it provided valuable data for elucidating the role of axial elongation in myopia development.

Yu et al. reported a detailed study of patients with congenital insensitivity to pain (CIP) associated with *PRDM12* mutations. They observed a range of manifestations, including pain insensitivity, self-mutilation, facial and limb defects, and recurrent infections. Remarkably, nearly every patient exhibited some degree of corneal injury, highlighting the significance of monitoring eye symptoms. The importance of early diagnosis and treatment were stressed, especially considering the challenge of managing corneal issues in CIP patients. While corneal transplantation is a potential treatment, it necessitates careful evaluation due to patients’ overall health and possible post-surgical complications. This research underscores the critical need for attentive eye care and customized treatments for individuals affected by *PRDM12*-related CIP. In their study, Chen et al. investigated the molecular basis of oculocutaneous albinism (OCA) in two families, aiming to facilitate prenatal diagnosis. They identified distinct mutations in the *TYR*, *TYRP1*, and *SLC45A2* genes. In one family, a patient exhibited OCA2 with Prader-Willi Syndrome due to a novel paternal deletion and a pathogenic mutation in the maternal chromosome. Prenatal diagnosis for this family revealed a carrier fetus. In another family, a patient was diagnosed with OCA2 and Angelman Syndrome due to a maternal deletion and a novel pathogenic mutation in the paternal chromosome. These findings expand our understanding of OCA-related mutations, emphasizing the importance of precise molecular classification for genetic counseling and early intervention in these cases.

Zhou et al. examined the pathogenic effects of *MYOC* gene mutations in patients with POAG. By analyzing specific myocilin (*MYOC*) variants, the research revealed genotype-phenotype links relating to the structure and function of this critical protein, including its secretion, subcellular localization, and potential role in autophagy and oxidative stress. The study found that secretion defects, often related to steric clash alterations, were closely linked to the pathogenicity of *MYOC* variants. Mutant forms of myocilin with reduced levels of secretion were found to accumulate in the endoplasmic reticulum, disrupting autophagy and increasing oxidative stress. The research provided an exciting example of how human genetics can lead to improved understanding of disease mechanisms, here shedding light on the pathophysiology of POAG.

A Chinese family with congenital retinoschisis was investigated using whole-exome sequencing and comprehensive clinical examination by Wang et al. A novel splice site mutation (RS1.c.53-1G>A) was identified in the *RS1* gene, suggesting potential for gene therapy and enhancing our understanding of this highly debilitating X-linked retinal disorder. Meanwhile, Tan et al. investigated two Chinese pedigrees with pigmentary glaucoma (PG). This revealed novel, compound heterozygous variants in the *CPAMD8* gene. The variants were classified as damaging or deleterious based on bioinformatics analysis. The autosomal recessive nature of disease transmission in these pedigrees and the strong evidence for a role of *CPAMD8* variants in pigment dispersion syndrome/pigmentary glaucoma were notable findings. Additionally, the study emphasized the need for further research to explore the role of *CPAMD8* in iris stromal abnormalities and immunological factors in the development of pigmentary glaucoma.

In a tour-de-force multiomics study, Jiang et al. explored the genetic determinants of corneal resistance factor (CRF), a key index of corneal biomechanics that is relevant to diseases including keratoconus and to ocular surgical procedures such as corneal transplantation. The researchers began by fine-mapping GWAS loci for CRF, which yielded credible sets comprising 181 signals. Leveraging gene expression data for non-eye tissues from the Genotype-Tissue Expression (GTEx) portal and single cell transcriptomics datasets from cornea, limbus and conjunctiva, the authors found that more than 25% of CRF and GTEx signals were shared. As well as indicating the potential involvement of corneal stromal cells and limbal cells, the research strongly suggested key roles for genes impacting extracellular matrix composition, mechanosensing, and signaling. Colocalization analysis of GWAS signals for CRF and keratoconus further strengthened the clinical relevance of the findings. This work emphasized the importance of considering the tissue-specific context in which genetic variants exert their effects, elegantly demonstrating how this approach can uncover potential therapeutic targets for corneal disorders.

